# Antitumor Effects of Umbelliprenin in a Mouse Model of Colorectal Cancer

**Published:** 2018

**Authors:** Mohamad Naderi Alizadeh, Mohsen Rashidi, Ahad Muhammadnejad, Taraneh Moeini Zanjani, Seyed Ali Ziai

**Affiliations:** a *Department of Pharmacology, school of medicine, Shahid Beheshti University of Medical Sciences, Tehran, Iran. *; b *Department of Physiology and Pharmacology, Faculty of Medicine, Mazandaran University of Medical Sciences, Sari, Iran. *; c *The Health of Plant and Livestock Products Research Center, Mazandaran University of Medical Sciences, Sari, Iran. *; d *Cancer Biology Research Center, Cancer Institute of Iran, Tehran University of Medical Sciences, Tehran, Iran.*

**Keywords:** Umbelliprenin, Colorectal cancer, cytotoxic effect, Anti-carcinogenic effect, Immunohistochemistry, CT26, Anti-angiogenesis

## Abstract

Umbelliprenin is a sesquiterpene coumarin with vitro anti-carcinogenic activities. The aim of this study was to investigate the antitumor effects of umbelliprenin in animal models of colorectal cancer. The cytotoxic effects of umbelliprenin were explored on CT26 and L929by MTT assay. In this study, colorectal tumors developed in mice by intradermal injection of CT26 cell line. Tumor size, serum levels of IFN-γ and IL-4 by ELISA, and Ki-67, MMP2, MMP9, VEGF and E-cadherin markers by IHC method were evaluated. The results showed that umbelliprenin inhibited the cancer cells in a concentration-dependent manner. IC50 Evaluation showed that L929 cells were more resistant to Umbelliprenin than CT26 cells. Umbelliprenin treatment in both tumor-bearing mice and control normal mice showed significantly increased IFN-γ and decreased IL-4(*P *< 0.05). The pathologic findings had shown that the E-cadherin marker in the umbelliprenin treated cancerous mice were significantly higher compared to the control group (*P *< 0.05) while the expression of Ki-67 marker was reduced significantly (*P *< 0.05). Markers involved in angiogenesis including VEGF, MMP2, and MMP-9 in the cancerous mice group treated with umbelliprenin showed a significant decrease compared to the control group (*P *< 0.05). Metastasis to lung and liver was reduced in umbelliprenin treated group. Our results showed that umbelliprenin inhibited CT26 tumor cells *in-vitro***. **The *in-vivo* reduction of tumor size, angiogenesis, and proliferation markers and the absence of metastasis represents the antitumor effects of umbelliprenin on colorectal cancer. The results showed that umbelliprenin can be considered as a good candidate for the treatment of colorectal cancer.

## Introduction

Colorectal cancer is the second reason of mortality in USA and also the third causing agent of mortalities related to cancer in Iran (1, 2). Umbelliprenin is considered as an anti-cancer agent with cytotoxic effects (3). Umbelliprenin is a sesequiterpene coumarin that is synthetized by different spices of *Ferula* of umbeliferace (4). Various studies have shown that umbelliprenin possess different biologic and pharmacologic characteristics such as anti-inflammatory, antioxidant, cytotoxic, antibacterial, antimalarial, anti-HIV, anti-leishmania, antihypertensive, anti-osteoporosis, and antiarrhythmic (5-8). Results of the new researches have shown that umbelliprenin causes inhibition of specific matrix metalloprotease and oxidosqualene cyclase activity and also has pro- apoptotic characteristics and anti-cancer effects (6, 9). This compound increases lymphocytes response to mitogens and induces immune system-related anti-tumor effects. Furthermore, possibly because of intervention with fibrinolytic system, umbelliprenin can affect angiogenesis and it decreases metastasis (3, 10). This study was designed to evaluate anti-tumor effects of umbelliprenin on an animal model of colorectal cancer. 

## Experimental

*MTT assay*

The CT26 and L929 cell lines (cell lines were purchased from Pasteur institute) were cultured in RPMI-1640 (Gibco®, Life Technologies, USA) medium supplemented with 10% fetal bovine serum (Gibco®, Life Technologies, USA), and 1% penicillin/streptomycin (100 U/mL) in a humidified atmosphere containing 5% CO2 and 95% air at 37 °C. The cells were seeded into 96-well culture plates at densities of 1 × 105 cells per well. After 24 h, they were treated with 3, 6.25, 12.5, 25, 50, 100 and 200 µg/mL umbelliprenin for 24, 48 and 72 h. After the treatment time passed, 10 µL of MTT solution was added to each well of 96-well plates and incubated for 4 h at 38 °C, then the purple MTT-formazan crystals were dissolved by adding 150 µL of DMSO. The absorbance of the samples were measured with ELISA reader at 540 nm.

*Colorectal cancer model and study groups*

Six to eight-weeks age BALB/c mice (Pasteur institute, Tehran, Iran) were divided into two main groups including tumor groups and non-tumor control groups. In tumor groups, CT26 cancer cells were injected subcutaneously (1 × 10^5^ cells/0.1 mL PBS/mouse) into the lower right flanks of each mice. After 17 days, when the tumors had reached an average volume of 400–500 mm^3^, the tumor-bearing BALB/c mice were intraperitoneally injected with umbelliprenin (pharmaceuticalgrade synthesized by research center of Mashhad University of Medical Sciences, Iran) 12.5 mg/kg/ 200µL liquid paraffin (group A, n = 6), liquid paraffin 200µL (group B, n = 6), and normal saline 200µL (group C, n = 6) daily for one week. Similar protocol was carried out on control mice with the injection of Umbelliprenin with liquid paraffin (group D, n = 6), liquid paraffin (group E, n = 6), and normal saline (group F, n = 6). This study was approved by Shahid Beheshti University of Medical Sciences Research Ethical Committee (IR. SBUM.RETECH.REC.1395.847).

*Tumor Volume calculation*

According to Khaghanzadeh* et al.*(11), tumor volume (mm^3^) was determined in tumor-bearing animals, on a 2-day intervals schedule, with a digital caliper in 12 to 34^th^ days after the injection. Tumor volume based on caliper measurements were calculated by Jensen *et al.* study formula (12):

Tumor volume* = *1/2*(*length* × *width^2^)

*Histopathological assay*

All mice, after anesthesia by co2, were sacrificed with cervical dislocation. Tissue samples as well as Liver, lung, and kidneys were collected from all animals, fixed in formalin and embedded in paraffin. Then, 5-µm cuts were prepared from each tissue block and were subjected to routine Hematoxylin and Eosin (H&E) staining. The stained slides were examined by light microscopy. Pathologic complete response (pCR) assessment was assumed, which measures response to dealing based on the amount of remaining tumor cells, as well as mitosis, necrosis, and pleomorphic rate. The pCR scoring follows as; R = 0, there is no response and no evidence regarding the reduced population of the malignant cells; R = 1, there is a partial-weak response and at least 30% of malignant cell fibrosis is observed; R = 2, there is a partial-moderate response and at least 70% of malignant cell fibrosis is observed; R = 3, there is a complete response and no presence of the malignant cells.

*Immunohistochemistry assay*

Formalin-fixed and paraffin-embedded tissue slides were also subjected to immunohistochemical assay of Ki-67(ab15580 Abcam, USA), CD31 (ab28364 Abcam, USA), VEGF (ab46154 Abcam, USA), MMP2 (ab37150 Abcam, USA), MMP9 (ab38898 Abcam, USA), and E-Cadherin (PM 170 AA Biocare, UK) using commercially available antibodies according to the manufacturers’ instructions, and being analyzed by an expert pathologist. Rate staining as (0) - no stained cells, (1) - stained cells <1/100, (2) - 1/100 ≤ stained cells < 1/10, (3) - 1/10 ≤ stained cells < 1/3, (4) - stained cells = 1/3 &< 2/3, (5) - stained cells > 2/3; Intensity as 0 = none, 1 = weak, 2 = intermediate, 3 = strong; and Allred score as 0–1 = no reactive, 2–3 = weak reactive, 4–6 = intermediate reactive, 7–8 = high reactive

*Determination of IFN-γ and IL-4*

Serum concentrations of IFN-γ (HRP, MABTECH, Sweden) and IL-4 were measured by enzyme-linked immunosorbent assay (ELISA) using specific kits (HRP, MABTECH, Sweden) according to the manufacturer’s guidelines. The o-phenylenediamine was used as chromogenic substrate for the horseradish peroxidase enzyme. The color intensity produced because of oxidative coupling reaction of the substrate and enzyme and was assessed at a wavelength of 492 nm, using an Anthos 2020 micro plate reader (Anthos, Wals, Austria).

*Data analysis*

The data were analyzed using the GraphPad Prism 4 ver. 4.03 software (GraphPad Software, La Jolla, CA). Data are presented as mean  ± SD. The differences in all data were assessed by one-way analysis of variance (ANOVA). Differences were considered statistically significant at *P *< 0.05.

## Results

*Inhibitory effects of umbelliprenin on cell lines*

**Figure 1:**
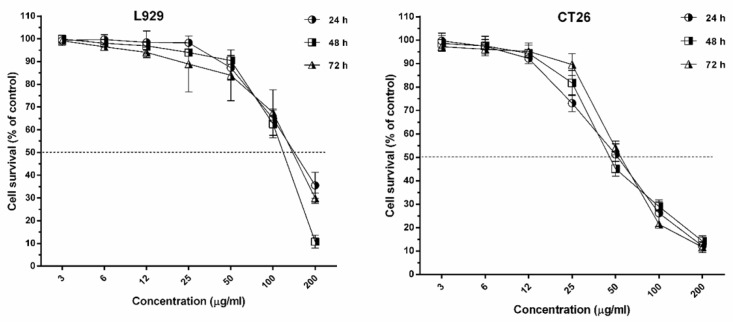
The survival rate of L929 and CT26 after treatment with umbelliprenin in 24, 48 and 72 h incubation times

The IC_50_ of umbelliprenin on cancer cell lines of CT26 and L929 in 24, 48, and 72 h of incubation is shown in Table 1 & Figure 1. Results showed that inhibitory effects of umbelliprenin in concentrations of 25 and 50 µg/mL were significantly different between incubation times (*P *< 0.05). The results showed that IC_50_ of L929 cells was three times greater than CT26 tumor cells.

**Table 1:** Mean IC_50_ ± SD (95% CI) of umbelliprenin for CT26 and L929 cell lines

**Cell line**	**24 h**	**48 h**	**72 h**
CT26	51.4 ± 2.9 (46-57.4)	53.2 ± 3.6 (46.6-60.8)	56.37 ± 2.5 (51.6-61.6)
L929	173.4 ± 2.9 (169-177.4)	134.2 ± 3.6 (140.6-128.8)	164.37 ± 2.5 (158.6-169.6)

**Table 2:** Histopathologic status of liver, lung and kidneys in normal mice

**Groups**	**Tissues**		
	**Liver (No. of mice)**	**Lung (No. of mice)**	**Kidney (No. of mice)**
Umbelliprenin (Group D)	Normal (5) Mild necrosis (1)	Normal (3) necrosis (1) Edema (2)	Normal (5) Edema (1)
Liquid paraffin (Group E)	Normal (2) Mild necrosis (2) Edema (1) Mild necrosis + Edema (1)	Normal (3) Edema (3)	Normal (4) Edema (1) Infiltrative cells + Edema (1)
Normal saline (Group F)	Normal (6)	Normal (4) Edema (1) Infiltrative cells (1)	Normal (6)

**Figure 2:**
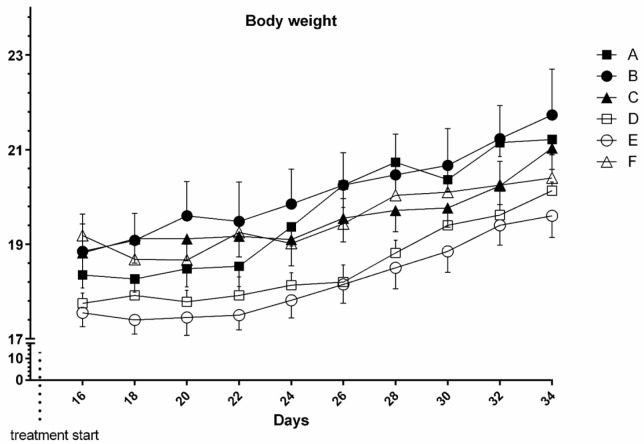
Mice body weights in study groups: Umbelliprenin (group A & D), liquid paraffin (group B & E) and saline (group C & F). Each point represents mean of 6 mice body weight ± S.E.M.

**Figure 3:**
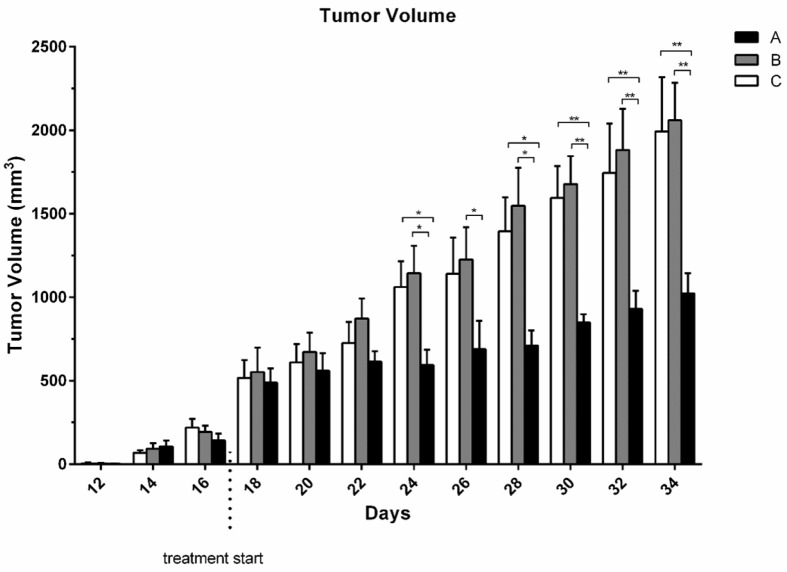
Tumor volume/Body weight ratio (TV/BW) in tumor bearing mice. Each bar represents mean of 6 mice ± S.E.M

**Figure 4:**
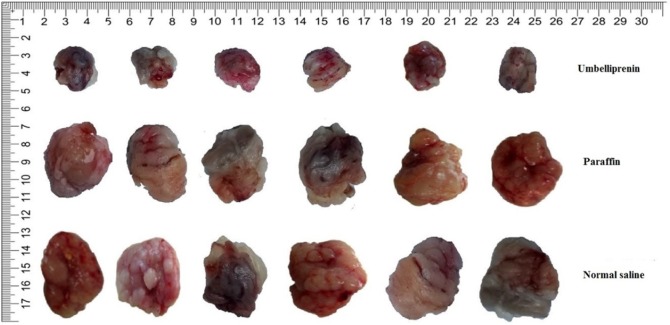
photographs of tumors extracted from the 6 mice at the end of study

**Figure 5:**
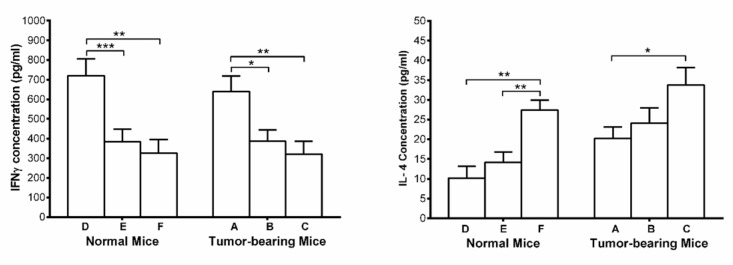
Mean of Serum concentration of IFN-γ and IL-4 in tumor-bearing groups (Umbelliprenin (A), liquid paraffin (B), and normal saline (C)) and normal mice (Umbelliprenin (D), liquid paraffin (E), and normal saline (F))

**Figure 6:**
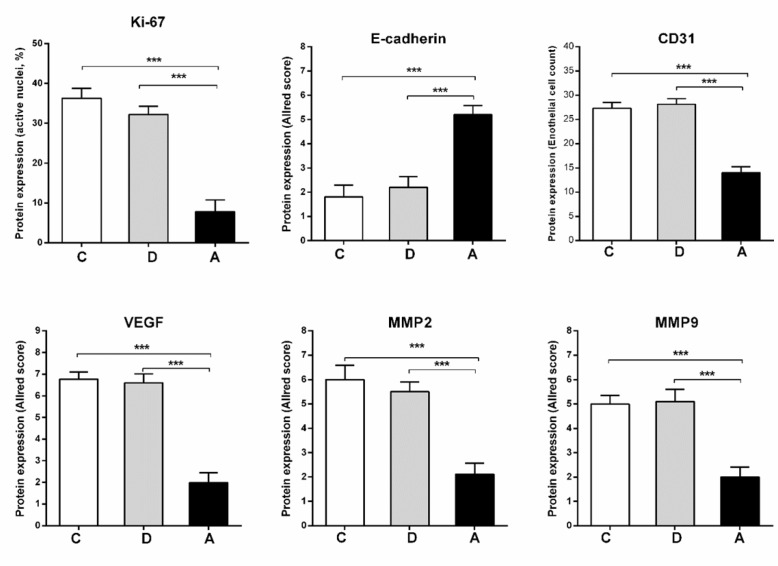
Immunohistochemical results of Ki-67, CD31, VEGF, MMP2, MMP9 and E-cadherin in tumor groups treated with umbelliprenin (A), liquid paraffin (B) or normal saline (C).

**Figure 7:**
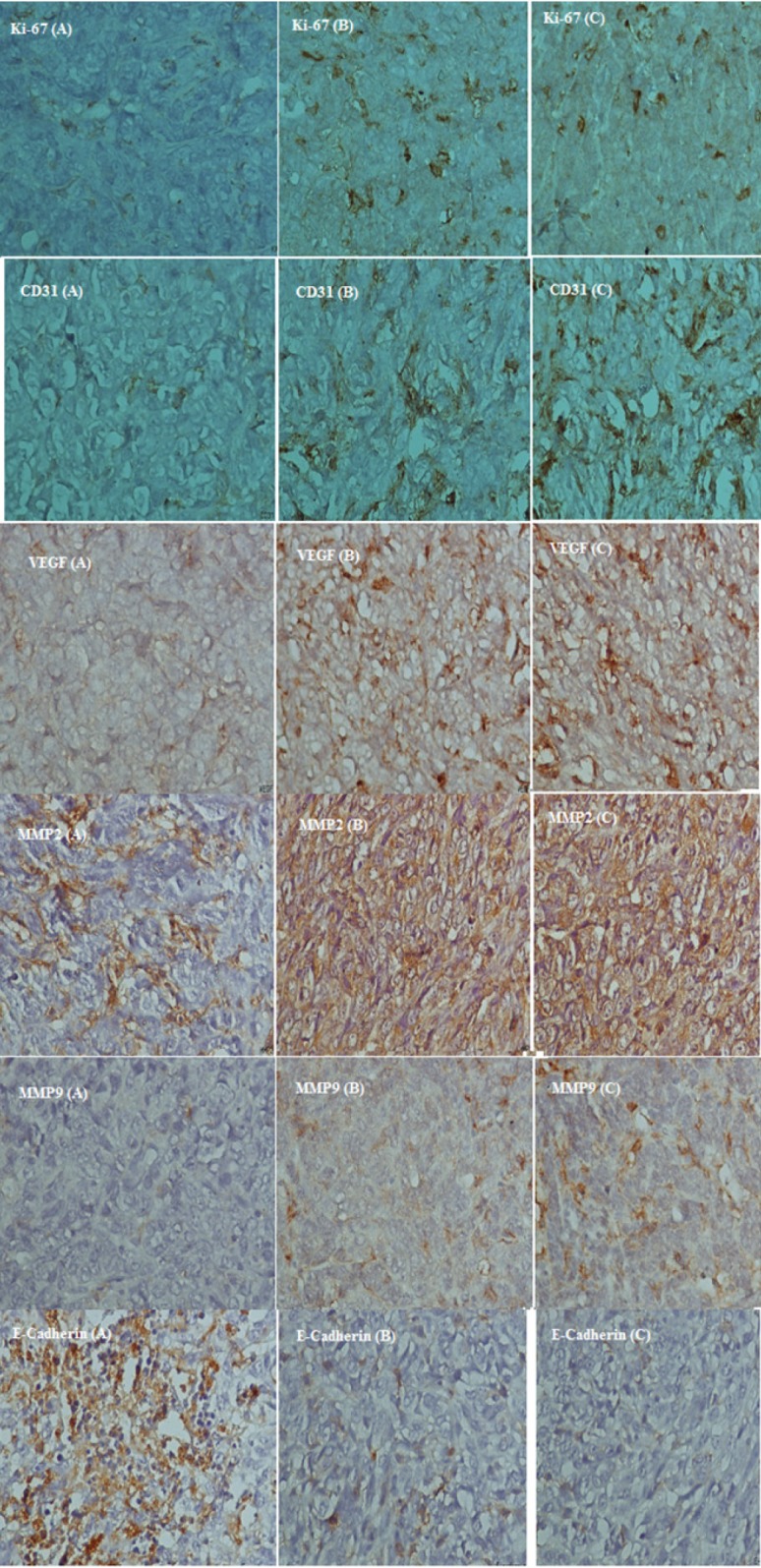
Photography of Immunohistochemical staining of Ki-67, CD31, VEGF, MMP2, MMP9 and E-cadherin in tumor-bearing mice

**Figure. 8:**
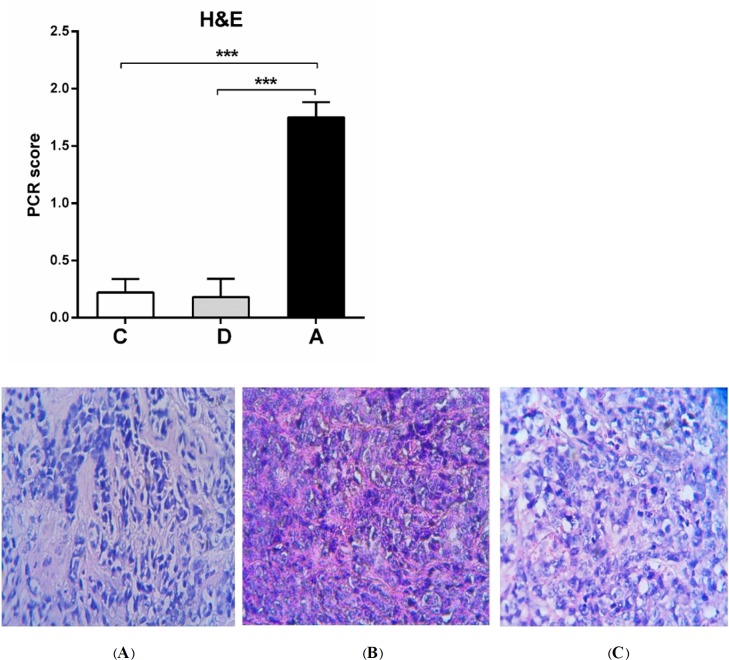
H&E staining of tumor groups. pCR scanning based on H&E stained samples of tumor-bearing mice showed significant tumor destruction by umbelliprenin (A) compared to liquid paraffin (B) and normal saline (C) groups.

*In-vivo effects of umbelliprenin*

*Body weight*

Results showed that body weight alteration between and within groups in time course of the study didn’t change significantly both in tumor bearing and normal mice (*P* > 0.05) (Figure 2).

*Tumor volume*

From 14^th^ day the tumor mass was measurable. Although there was no significant difference between group receiving umbelliprenin and control group and liquid paraffin group up to day 22; however, the mean TV/BW in umbelliprenin group in days 24, 26, 30, and 32 were significantly decreased compared to control and liquid paraffin group. There was no significant difference for mean TV/BW between any liquid paraffin and control groups in any day (*P *> 0.05) (Figures 3 & 4)

*Serum IFN-γ and IL-4*

Serum concentration of IFN-γ, showed significant increase in umbelliprenin group compared to control group and liquid paraffin group in both normal and tumor bearing mice. On the other hand, serum concentrations of IL-4 showed significant decrease in both tumor and normal mice on umbelliprenin compared to control group and liquid paraffin group (Figure 5).

*Immunohistochemistry*

Proportion of active nuclei indicated by Ki-67 expression, was decreased significantly in umbelliprenin group (A) compared to liquid paraffin (B) and normal saline (C) group (*P *< 0.001). Expression of CD31, VEGF, MMP2, and MMP9 markers in umbelliprenin group (A) showed significant decrease compared to liquid paraffin (B) and normal saline (C) group (*P* < 0.05). Also, the expression rate of E-cadherin in umbelliprenin (A) group was significantly increased compared to normal saline (C) and liquid paraffin (B) groups (*P *< 0.001) (Figures 6 & 7).

*Histopathology results*

The average of pCR and percentage of tumor cells destroyed in umbelliprenin group was significantly higher compared to normal saline (C) and liquid paraffin group (B) (*P *< 0.001). (Figure 8).

*Evaluation of metastasis to liver, lung and kidneys*

No signs of metastasis to kidneys observed in any tumor-bearing mice groups. There were no metastasis sings in liver, and lung in umbelliprenin group, but, three metastasis cases to liver and four metastasis cases to lung were observed in liquid paraffin group, and two metastasis cases to liver and 6 cases to lung were observed in normal saline group. 

*Effects of umbelliprenin on the liver, lung and kidneys in normal mice*

Umbelliprenin in normal (non-tumor) mice had some effects (Table 2).

## Discussion

Colorectal cancer after stomach and esophagus cancer, is the most prevalent gastro-intestinal cancer in Iran (13). Results of the present study showed that umbelliprenin has concentration-dependent toxic effects against CT26 cancer cell line. The evaluation of IC_50_ in the studied cell lines showed that the toxic effects of umbelliprenin on L929 cell line was more than CT26 cell line in all three incubation times. 

Until now, the cytotoxic effects of umbelliprenin on different cancer cell lines were evaluated in different studies. The results of Khaghanzadeh *et al.* showed that umbelliprenin has inhibitory activity against adenocarcinoma and lung cancer cells at low concentrations, but has no toxic activity against PBMCs (11). Barthomeuf *et al*. found that cytotoxic activity of umbelliprenin leads to inhibition of cancer cell lines of melanoma, lungs, prostate, ovaries, breasts, and colon. They find that high toxicity of umbelliprenin on M4Beu cells is mediated by inhibition of G1 in cell cycle and apoptosis induction via caspase cascade, and also low cytotoxicity on primary fibroblasts (9). In Rashidi *et al*. study, it has been determined that umbelliprenin has the potential of inhibitory activity against tumor cells (14). Furthermore, it was found that the inhibitory activity of umbelliprenin is significantly higher against breast cancer cells (4T1 and MCF-7) (14). This study also showed that umbelliprenin at cytotoxic concentrations could not inhibit normal cells (14). Gholami *et al*. demonstrated that umbelliprenin, by activation of caspase 8 and 9, activates internal and external apoptosis pathways respectively in Jurkat T-CLL. Moreover, they showed that umbelliprenin promotes apoptosis process by inhibition of 

Bcl-2 (15). 

In the present study, the mean TV/BW in umbelliprenin group was significantly decreased in days 24, 26, 30, and 32 compared to normal saline and liquid paraffin groups. Generally, our findings showed that umbelliprenin can be effective in reduction of tumor size. These findings were consistent with Iranshahi *et al*. (9) and also Khaghanzadeh *et al*. studies (6, 11) Probably by the induction of apoptosis mechanisms noted above.

Angiogenesis in various tumors such as colorectal cancer is mediated by different molecules such as IL-1β, bFGF, VEGF, MMPs, and TNFα. Oncologic alterations of tumor cells can play role via angiogenic factors in induction and development of angiogenesis (16, 17). In the present study, IHC method was used to evaluate angiogenesis and metastasis in tumor tissues by MMP2, MMP9, VEGF, and CD31 markers assay. The results of this study showed significant decrease in expression of VEGF, MMP2, MMP9, and CD31 factors in tumor-bearing mice under treatment with umbelliprenin compared to normal saline group. Furthermore, in our study, there were no signs of metastasis to liver, lungs, and kidneys in the umbelliprenin group. The results of our study indicates the important role of umbelliprenin in inhibition of metastasis of colorectal cancer cells especially to liver and lung. Studies have shown that inhibition or reduction of VEGF, MMP2, MMP9, and CD31 activity leads to reduction of angiogenesis, invasion, and cancer cell metastasis (18-22). It seems that umbelliprenin by decreasing expression of these factors leads to less metastasis, although the mechanism details need to be understood. 

On the other hand, the expression of E-cadherin marker in umbelliprenin group was significantly increased compared to normal saline and liquid paraffin groups. E-cadherin is a membrane protein with essential role in cell-cell connection, and its decrease leads to unclenching of cancer cells and increases the possibility of metastasis (23, 24). It seems that the increasing of E-cadherin expression under the influence of umbelliprenin leads to stability of cell connections and probably inhibition of expression of metastatic factors induced by beta-cadherin.

Also, the results showed that expression of Ki-67 marker in tumor-bearing mice treated with umbelliprenin was significantly decreased. The decrease of Ki-67 via umbelliprenin can lead to reduction and inhibition of cell deviation which is one of the most important characteristics of cancer cells, and this process also can be effective in reduction of cancer cells proliferation (25). 

Furthermore, in the present study, we evaluated the necrosis rate and presence of malignant cells (mitosis and polymorphism) by using H&E staining and application of pathologic complete response system (pCR). The average of pCR and percentage of destroyed tumor cells in umbelliprenin group were significantly more than normal saline and liquid paraffin groups. 

In the present study, umbelliprenin significantly increased IFN-γ and decreased IL-4. Increasing of IFN-γ leads to activation of immune system against cancer cells and therefore induction of apoptosis in them (26). Studies have shown that IL-4 can lead to regulation of anti-tumor immune responses. The reduction of IL-4 levels in mice treated with umbelliprenin can help the anti-tumor activity of umbelliprenin. These results are consistent with findings of other researchers, as with Khaghanzadeh *et al*. (11). However, in our study, umbelliprenin increased levels of IFN-γ and decreased IL-4 both in tumor and non-tumor animals. These changes may be related to the direct effects of umbelliprenin on immune system, and needs more investigation. 

## Conclusion

Our results showed selective cytotoxicity of umbelliprenin on CT26 cells relative to L929 ones. Furthermore, umbelliprenin treatment in animal model of colorectal cancer showed reduction of tumor size, angiogenesis (showed by VEGF, MMP2, MMP9, and CD31 reduction and E-cadherin increment), proliferation (showed by KI-67 marker), and metastasis (showed by MMP2, MMP9 reduction and also no signs of liver, lung, and kidney metastasis). Umbelliprenin also potentiates immune response by IFN-γ increment and IL-4 decrease. So it is a good candidate for further investigations.
